# Biological Polymers: Evolution, Function, and Significance

**DOI:** 10.1021/acs.accounts.4c00546

**Published:** 2025-02-05

**Authors:** Kavita Matange, Eliav Marland, Moran Frenkel-Pinter, Loren Dean Williams

**Affiliations:** ^†^NASA iCOOL (Center for the Integration of the Origins of Life), ^‡^School of Chemistry and Biochemistry, Georgia Institute of Technology, Atlanta, Georgia 30332-0400, United States; § Institute of Chemistry, The Hebrew University of Jerusalem, Jerusalem 91904, Israel; ∥ The Center for Nanoscience and Nanotechnology, The Hebrew University of Jerusalem, Jerusalem 9190401, Israel

## Abstract

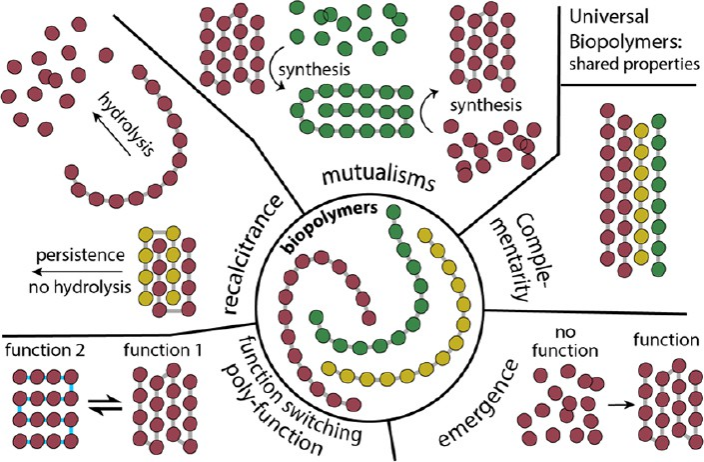

A holistic description of biopolymers and their evolutionary origins will contribute to our understanding of biochemistry, biology, the origins of life, and signatures of life outside our planet. While biopolymer sequences evolve through known Darwinian processes, the origins of the backbones of polypeptides, polynucleotides, and polyglycans are less certain. We frame this topic through two questions: (i) Do the characteristics of biopolymer backbones indicate evolutionary origins? (ii) Are there reasonable mechanistic models of such pre-Darwinian evolutionary processes? To address these questions, we have established criteria to distinguish chemical species produced by evolutionary mechanisms from those formed by nonevolutionary physical, chemical, or geological processes. We compile and evaluate properties shared by all biopolymer backbones rather than isolating a single type. Polypeptide, polynucleotide, and polyglycan backbones are kinetically trapped and thermodynamically unstable in aqueous media. Each biopolymer forms a variety of elaborate assemblies with diverse functions, a phenomenon we call polyfunction. Each backbone changes structure and function upon subtle chemical changes such as the reduction of ribose or a change in the linkage site or stereochemistry of polymerized glucose, a phenomenon we call function-switching. Biopolymers display homo- and heterocomplementarity, enabling atomic-level control of structure and function. Biopolymer backbones access recalcitrant states, where assembly modulates kinetics and thermodynamics of hydrolysis. Biopolymers are emergent; the properties of biological building blocks change significantly upon polymerization. In cells, biopolymers compose mutualistic networks; a cell is an Amazon Jungle of molecules. We conclude that biopolymer backbones exhibit hallmarks of evolution. Neither chemical, physical, nor geological processes can produce molecules consistent with observations. We are faced with the paradox that Darwinian evolution relies on evolved backbones but cannot alter biopolymer backbones. This Darwinian constraint is underlined by the observation that across the tree of life, ribosomes are everywhere and always have been composed of RNA and protein. Our data suggest that chemical species on the Hadean Earth underwent non-Darwinian coevolution driven in part by hydrolytic stress, ultimately leading to biopolymer backbones. We argue that highly evolved biopolymer backbones facilitated a seamless transition from chemical to Darwinian evolution. This model challenges convention, where backbones are products of direct prebiotic synthesis. In conventional models, biopolymer backbones retain vestiges of prebiotic chemistry. Our findings, however, align with models where chemical species underwent iterative and recursive sculpting, selection, and exaptation. This model supports Orgel’s “gloomy” prediction that modern biochemistry has discarded vestiges of prebiotic chemistry. But there is hope. We believe an understanding of biopolymer origins will progress during the challenging and exciting integration of chemical sciences and evolutionary theory. These efforts can provide new perspectives on pre-Darwinian mechanisms and can deepen our understanding of evolution and of chemical sciences. Our working definition of chemical evolution is continuous chemical change with exploration of new chemical spaces and avoidance of equilibrium. In alignment with our model, we observe chemical evolution in complex mixtures undergoing wet–dry cycling, which does appear to undergo continuous chemical change and exploration of new chemical spaces while avoiding equilibrium.

## Key References

- Frenkel-PinterM.; HaynesJ. W.; MohyeldinA.M.; CM.; SargonA. B.; PetrovA. S.; KrishnamurthyR.; HudN. V.; WilliamsL. D.; LemanL. J.Mutually stabilizing interactions between proto-peptides and RNA. Nat. Commun.2020, 111, 313710.1038/s41467-020-16891-5PMC730522432561731.^1^*This manuscript shows that cationic proto-peptides associate with RNA resulting in increased stability and persistence*. *The findings lend support to a coevolutionary history of biopolymer types*.
- RunnelsC. M.; LanierK. A.; WilliamsJ. K.; BowmanJ. C.; PetrovA. S.; HudN. V.; WilliamsL. D.Folding, Assembly and Persistence: The Essential Nature and Origins of Biopolymers. J. Mol. Evo.2018, 86, 598–61010.1007/s00239-018-9876-2PMC626770430456440.^2^*This manuscript evaluates universal as well as idiosyncratic characteristics of biopolymer types and incorporates this information into a model to explain their origins, selection and evolution*.
- Guth-MetzlerR.; MohamedA. M.; CowanE. T.; HenningA.; ItoC.; Frenkel-PinterM.; WartellR. M.; GlassJ. B.; WilliamsL. D.Goldilocks and RNA: Where Mg^2+^ Concentration Is Just Right. Nucleic Acid Res.2023, 51, 3529–35336987860 10.1093/nar/gkad124PMC10164553.^3^*This work describes and validates a Goldilocks model of RNA recalcitrance that explains how lifetime landscapes are modulated by RNA folding*.
- EdriR.; FisherS.; Menor-SalvanC.; WilliamsL. D.; Frenkel-PinterM.Assembly-driven protection from hydrolysis as key selective force during chemical evolution. FEBS letters2023, 597 ( (23), ), 2879–289637884438 10.1002/1873-3468.14766.^4^*This manuscript describes the influence of biopolymer assembly on hydrolysis rates and suggests that assembly was crucial for selection during chemical evolution*. *The generality of recalcitrance and its relationship with assembly is documented for all universal biopolymer types*.
- MatangeK.; RajaeiV.; Capera-AragonèsP.; CostnerJ. T.; RobertsonA.; KimJ. S.; PetrovA. S.; BowmanJ. C.; WilliamsL. D.; Frenkel PinterM.Evolution of Complex Chemical Mixtures Reveals Combinatorial Compression and Population Synchronicity. Nat. Chem.2024, in press10.1038/s41557-025-01734-x39939341.^5^*This work establishes an experimental model of chemical evolution using water as a chemical reactant, product and medium*. *It demonstrates that systems that can undergo continuous change while exploring new chemical spaces, and supports non-Darwinian evolution models of the Origins of Life*.

## Introduction

Around four billion years ago, prebiotic chemistry established the molecular keystones of biology, paving a path to life. Chemical and geological processes on the ancient Earth caused increases in the complexity of organic molecules, leading ultimately to the creation of RNA, DNA, protein, polysaccharides, bilayer-forming amphipaths, and the roots of biology.

The transition from small prebiotic chemical species to complex biological polymers presents some of the most fascinating and challenging questions in chemical and biological sciences. We propose that humankind will eventually understand, replicate, and technologically harness chemical progressions analogous to those that led to the formation of biopolymers on ancient Earth. This understanding will arise at the intersection of chemical sciences and evolutionary theory, ushering advancements in both fields. In this paper, we explore the nature and utility of this integration, explaining why it is essential for a comprehensive understanding of the past, present and future of biochemistry.

## Origins of Biopolymer Backbones

The evolution of biopolymer sequences follows reasonably well-understood Darwinian processes. Here we address a different issue, which is the evolution of backbones of polypeptide, polynucleotide, and polyglycan. We divide the big question of biopolymer backbone evolution into two distinct sub-questions. (i) Do the properties and behaviors of backbones suggest that they were created by evolutionary processes? (ii) Are there reasonable and defensible mechanistic models of those evolutionary processes? The first part of this manuscript deals with the first question and the second part deals with the second question. Over some years we and others^6−8^ have worked to understand the possibilities and potential of evolutionary creation of biopolymer backbones.

We show that products of evolution have distinctive and recognizable properties and behaviors, which we call footprints of evolution. Distinctions between evolutionary and nonevolutionary products apply across scale. Organisms, organs, organelles, molecular assemblies, and biological molecules are distinguishable from products of nonevolutionary physical, chemical, or geological processes. Recognizing biopolymer backbones as products of evolution provides a basis for understanding their current behaviors and their origins. The distinction between evolutionary and nonevolutionary molecular products can assist with NASA efforts to observe biosignatures beyond our planet. We start by enumerating the characteristics of known products of evolution, the brain and the ribosome, and compare those characteristics to those of biopolymer backbones.

The brain is a product of evolution. The brain has function–to integrate and store information and to organize organismal actions and responses through transmission of electrical and chemical signals. The brain is fragile. The human brain is composed of nearly 90 billion neurons with precise spatial organization and functions.^9^ The structure of the human brain is slowly being unraveled, allowing us to understand its functions.^10^

The ribosome is a product of evolution. The ribosome has function - to read mRNA and synthesize coded protein. The ribosome is fragile. The structure of the ribosome is directly related to its functions.^11−13^ The ribosome is a molecular machine of hundreds of thousands of atoms in precise locations in 3D space,^14^ comprising a peptidyl transferase center, a decoding center, and a polypeptide exit tunnel.

The brain and the ribosome are imprinted with evolutionary footprints. These footprints provide evidence of evolutionary origins and information on evolutionary histories. Although the scale is molecular, we can ask whether analogous information is available within biopolymer backbones. Do biopolymer backbones display footprints of evolution? Yes, they do. Molecular footprints of evolution are defined and discussed in detail in the narrative below. Non-evolutionary chemical and physical and geological processes do not leave evolutionary footprints. Interstellar polycyclic aromatic hydrocarbons are not fragile, do not have function, and are not imprinted with footprints of evolution.

## Molecular Footprints of Evolution

A function is conventionally described as an activity that contributes to organismal fitness. To understand molecules, we extend that definition to say that molecular function contributes to molecular fitness, which directly or indirectly enables molecular persistence. Biopolymer backbones are so intensely functional that they have persisted on Earth, unchanged, for around 4 billion years.

Biopolymers are based on long, organic backbones synthesized by condensation–dehydration chemistry via phosphorylated intermediates.^2^ Biopolymers are fragile, meaning that they are thermodynamically unstable and kinetically trapped.

Footprints of evolution are found in shared biopolymer properties including;

(i) Polyfunction and Function Switching,
(ii) Complementarity and Self-complementarity,
(iii) Recalcitrance: Intrinsic and Extrinsic Control of Chemical Fragility,
(iv) Molecular Mutualisms, and
(v) Emergence.

We believe these concepts, some of which we have invented or appropriated, and some of which are well-developed in the literature, have explanatory power for biochemistry and biophysics in general. Each of these terms is described in detail in the following narrative.

## Polyfunction and Function Switching

What is polyfunction? Polyfunction is access to broad landscapes of function. Polyfunction arises from untold iterations of evolutionary selection, exaptation, reselection, and re-exaptation. For example, ancestors of human metacarpus and phalanges (hands) were recursively selected/exapted for a variety of functions before they were selected for propulsion and stability in water (as fish fins), after which they were selected for terrestrial quadrupedal locomotion, then for climbing, grasping, communication, tactile exploration, etc. This long chain of recursive selection/exaptation leads to polyfunction. The function of human hands is simply to be broadly functional. Human hands have utility in boxing, writing, driving, swiping left, etc. These functions extend beyond those specifically selected during evolution.

**Figure 1:**
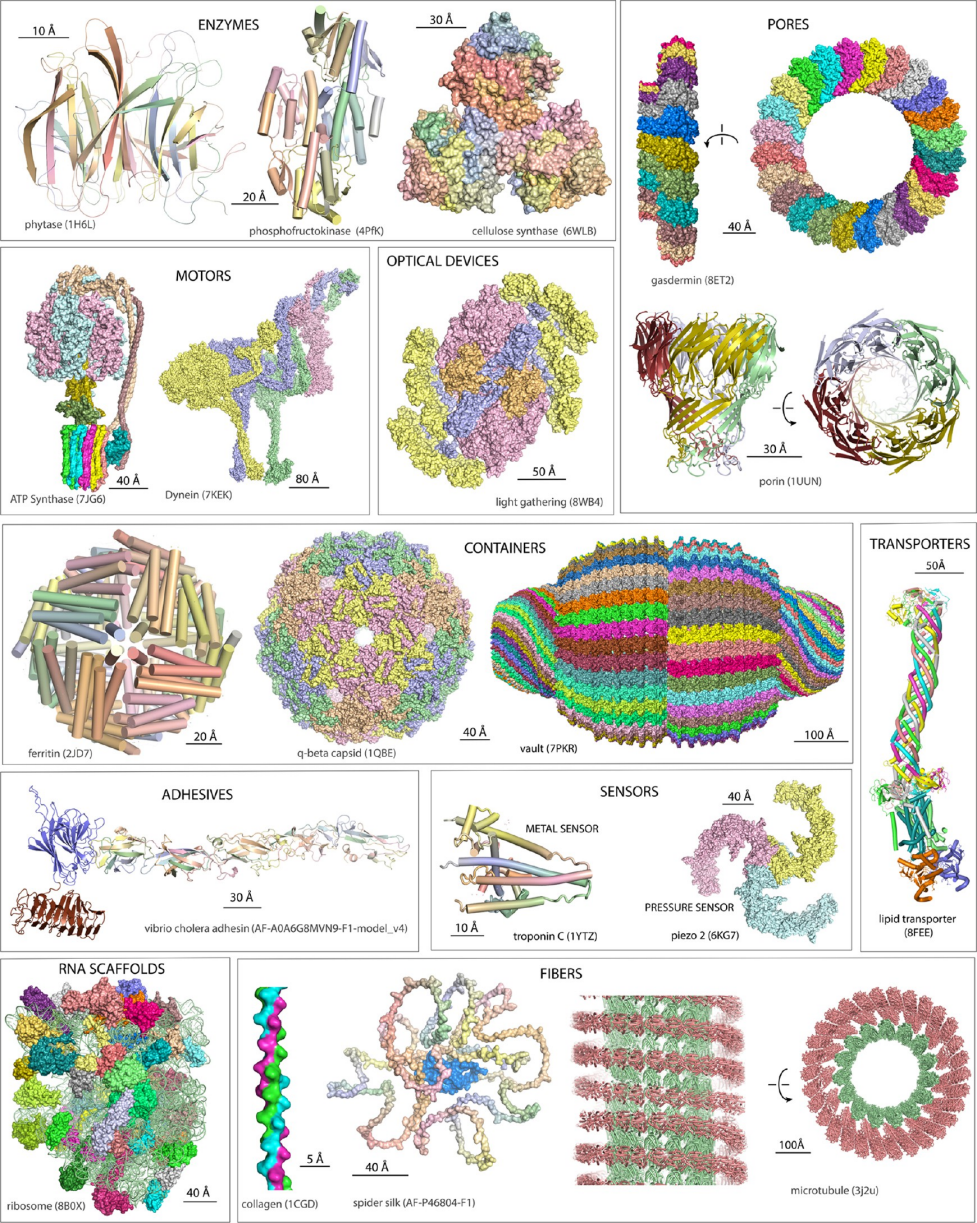
Structures and functions of polypeptides. Polypeptides are polyfunctional, with access to a seemingly infinite landscape of functional space. Each of these structures is based primarily on self-assembly of the polypeptide backbone, which is self-complementary. Amino acid sequence is a second order perturbation of backbone-based assembly. Coordinates were obtained from the PDB or the AlphaFold database and were visualized with PyMol. Polyfunction is consistent with evolutionary origins of the backbone.

**Figure 2:**
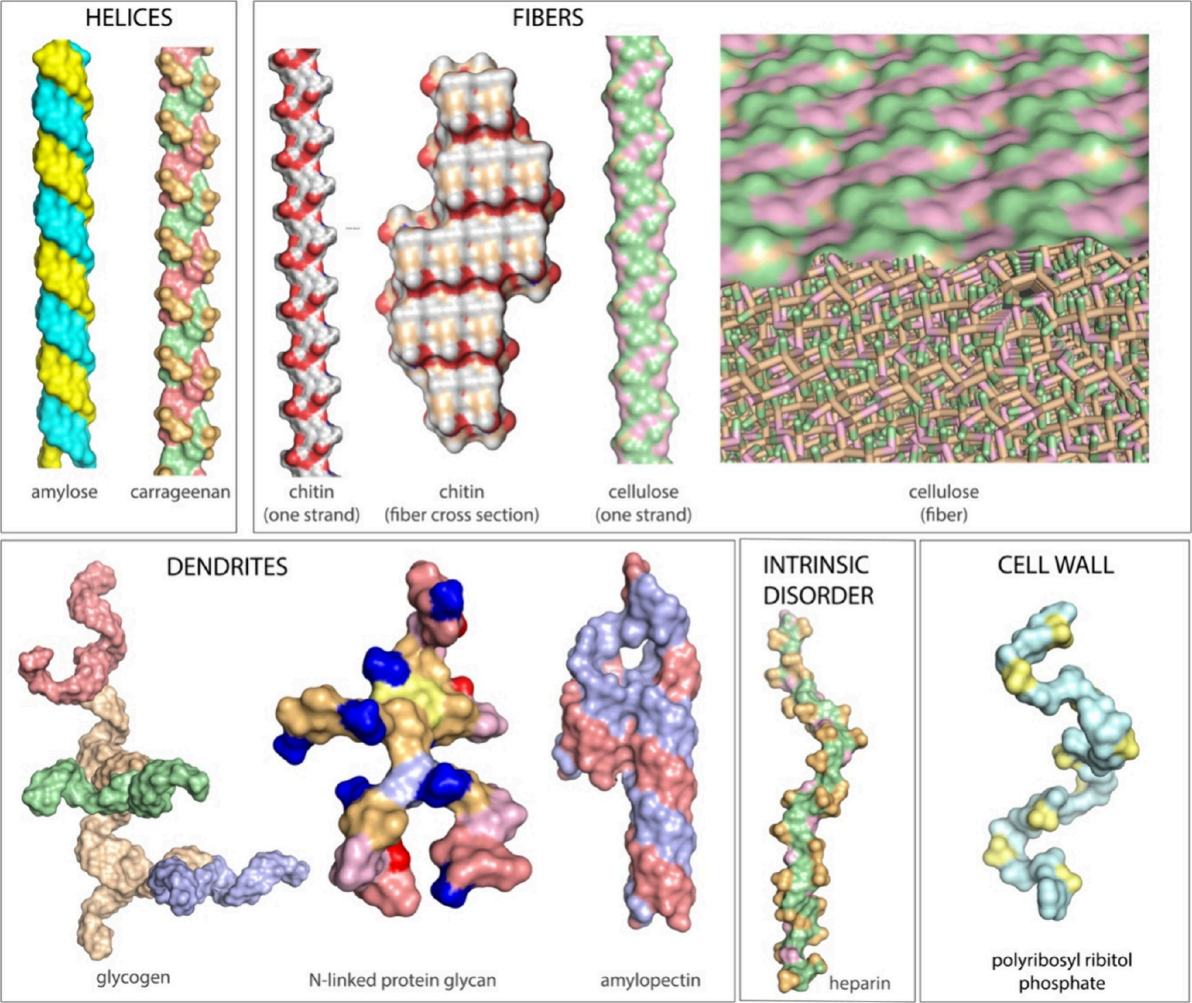
Structures and functions of polyglycans. Polyglycan is polyfunctional, with access to incredibly broad landscapes of function. Polyglycan assemblies are based primarily on self-complementarity of glucose. Coordinates were obtained from various databases and were visualized with PyMol.

Biopolymers, like human hands, are polyfunctional (Figures 1 and 2). Polypeptide (Figure 1) can be intrinsically disordered and can form α-helical, β-sheet and mixed α/β globular enzymes,^15^ and a broad variety of fibers,^16^ motors,^17^ containers,^18^ transporters,^19^ sensors,^20^ and signals,^21^ optical devices,^22−24^ adhesives,^25^ pores,^26^ brushes,^27^ and pumps.^28^ Globular enzymes have insides and outsides - solvent-accessible surfaces and solvent-shielded interiors. The interiors are ideal for functions such as catalysis of organic reactions. Polynucleotide has an expansive array of functions and is informational,^29^ catalytic,^30,31^ and structural.^32^ Polysaccharide has a broad array of functions and can form single, double, or triple helices,^33,34^ worm-like chains,^35^ cell walls,^36^ insoluble fibers that are chemically robust,^37^ and soluble dendrites^38^ (Figure 2) that can hydrolyze quickly and release chemical energy on demand. Each type of biopolymer backbone is polyfunctional.

A general characteristic of biopolymer backbones that contributes to polyfunction is the capacity to fundamentally remodel structural and functional landscapes via extremely subtle chemical changes. Insertion of prolines into a polypeptide abolishes the ability to form α-helices or β-sheets and tips structure toward noncatalytic collagen-type assemblies.^39^ Conversion of polyalanine to polyglycine converts α-helix to intrinsic disorder.^40^ Removing one atom from the RNA backbone to form the DNA backbone changes assembly states, helical form, hydrolytic lifetime, and catalytic potential.^15^ Changing the anomeric linkage of polyglucose from β(1,4) to α(1,4) changes the assembly state, hydrolytic lifetime, and functions. This minor chemical change converts cellulose^37^ to amylose.^34^ Introducing 10% (1,6) cross-links coverts amylose to glycogen.^38^

In sum, biopolymers have polyfunction and proficiency to remodel functional landscapes through subtle chemical changes. Chemical species produced by nonevolutionary processes do not have function or polyfunction and do not undergo function-switching. Polyfunctionality and function switching cannot be explained by mechanisms other than origins by evolution.

## Complementarity and Self-complementarity

Molecular complementarity within and between biopolymers contributes to fine control of structure and function. The polypeptide backbone is intrinsically self-complementary, as seen in the matched hydrogen bonding donor/acceptor arrays of α-helices or β-sheets.^2^ Polyglucose is self-complementary, as seen in assemblies of amylose,^34^ cellulose,^37^ and many other assemblies (Figure 2). The side chains of DNA and RNA are complementary as seen in duplex DNA and structural RNAs.^15^

**Figure 3:**
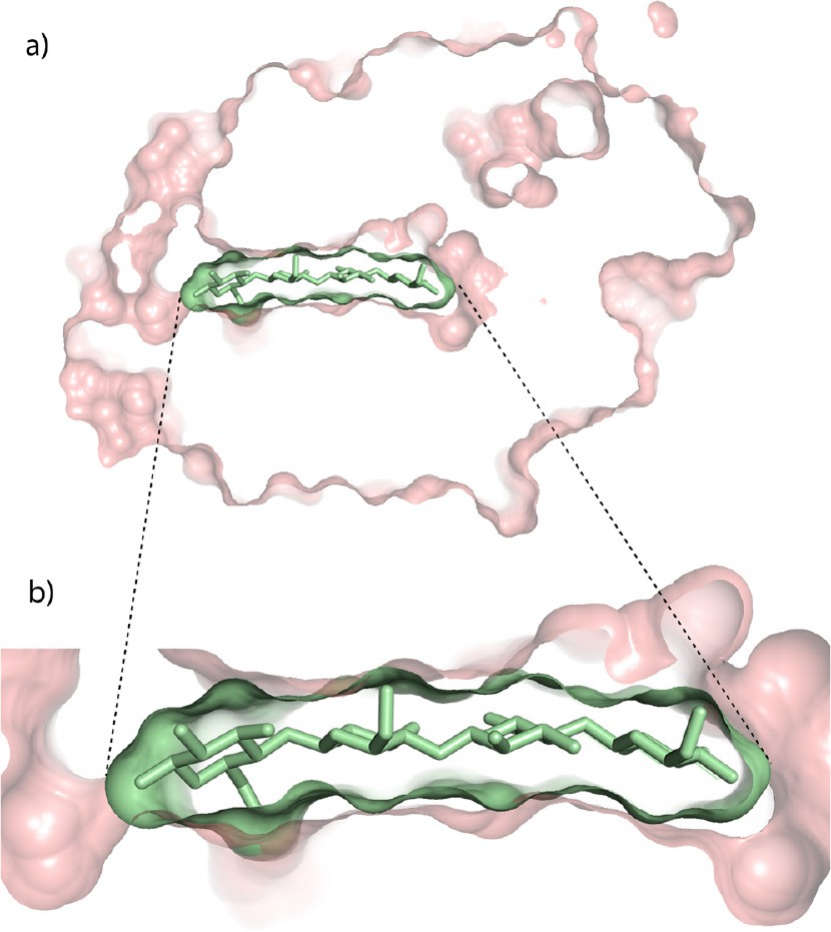
Complementary molecular interactions between the protein cellobiohydrolase I (pink) and the saccharide β(1–4) tetraglucose (green). Van der Waals surfaces are indicated. (a) A slice through the entire complex. (b) A zoomed view into the complentary protein-glycan interface (PDB entry5cel).

Biopolymers are heterocomplementary. Proteins can specifically recognize and bind to proteins, DNA or RNA, polyglycans, and small molecules. An example of complementarity of protein and polysaccharide is seen in Figure 3. The broad competence in self- and heterocomplementarity is not seen in nonbiological organic polymers and is consistent with coevolutionary origins.

## Recalcitrance: Intrinsic and Extrinsic Control of Chemical Fragility

**Figure 4:**
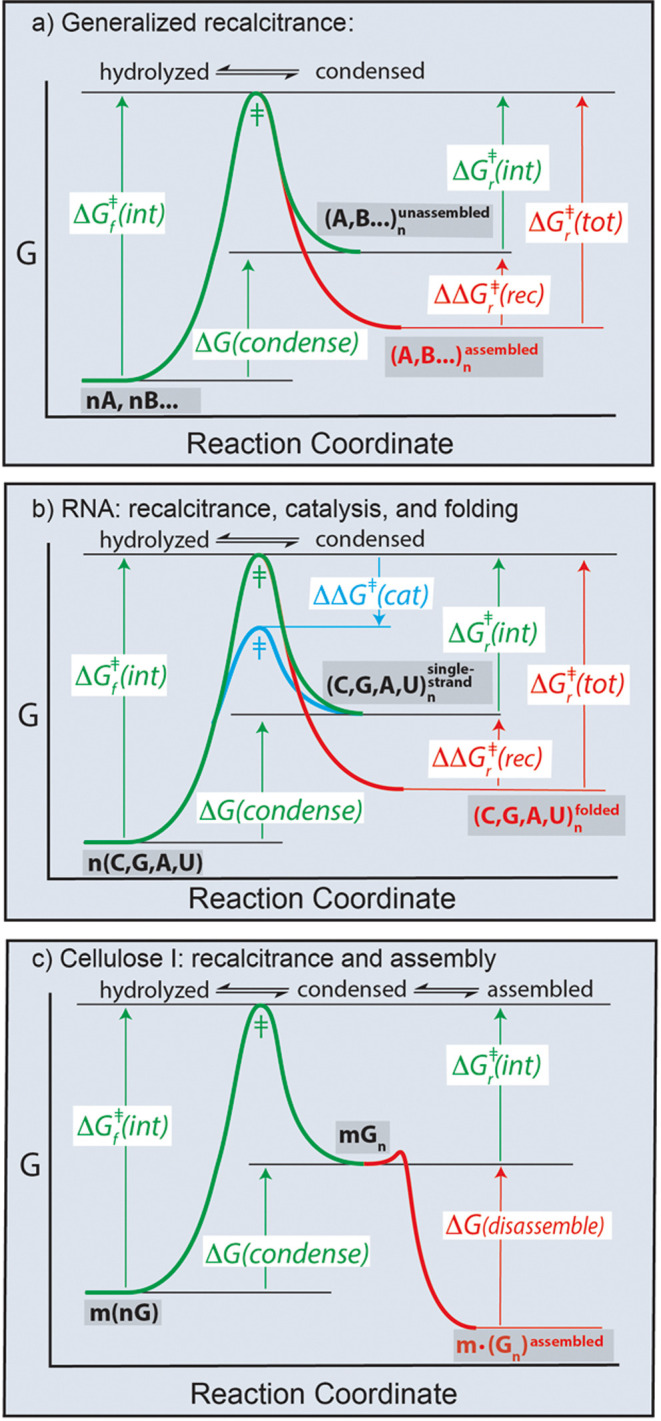
Assembly renders biopolymers recalcitrant, with abilities to persist in aqueous environments far longer than predicted by intrinsic chemical lifetimes. (a) A generalized reaction coordinate illustrating recalcitrance. The activation energy for hydrolysis of an assembled biopolymer is greater than for an unassembled biopolymer. The free energy of condensation of monomeric nucleotides (nA, nB, ...) to form polymers (A, B, ...)_*n*_ in aqueous media is positive [Δ*G* (*condense*) > 0]. Intrinsic activation free energies for condensation [Δ*G*_(*f*)_^‡^ (*int*)] and hydrolysis [Δ*G*_(*r*)_^‡^ (*int*)] are green. The total activation energy for hydrolysis of the assembled state Δ*G*_*r*_^‡^ (*tot*) is greater than in the unassembled state by ΔΔ*G*_*r*_^‡^ (*rec*). Both of these parameters are red. The *f* indicates the forward reaction (condensation dehyration) and *r* indicates the reverse reaction (hydrolysis). In this scenario, hydrolysis occurs in both the assembled or unassembled state but at different rates. (b) A catalyst or enzyme decreases the activation energies of condensation and hydrolysis by ΔΔ*G*^‡^ (*cat*). Assembly causes the activation energy of hydrolysis to increase by ΔΔ*G*_*r*_^‡^ (*rec*). (c) Cellulose 1 does not hydrolyze in the assembled state. The total activation free energy for hydrolysis is the sum of the intrinsic activation free energy of hydrolysis plus the free energy of disassembly (decrystallization).

Evolution produced fragile polymers^41−48^ that, paradoxically, dominate much of Earth’s chemistry. Biopolymers are large, complex, and fragile (thermodynamically unstable and kinetically trapped). Biopolymers degrade spontaneously in aqueous media.^41−48^ The negative free energy of hydrolysis (positive free energy for condensation–dehydration, Δ*G* (*condense*) > 0) is illustrated in Figure 4. Given sufficient time, DNA, RNA, polypeptide, and polyglycans degrade in water into small monomeric building blocks. Biopolymers persist in part because of kinetic trapping. Building blocks are linked by bonds that have high intrinsic activation energies of hydrolysis, as indicated by Δ*G*_(*r*)_^‡^ (*int*) in Figure 4. Kinetically trapped bonds include phosphodiester, peptide, and glycosidic bonds.^41−48^

One of the most astounding proficiencies of biopolymers is their ability control their own destinies by manipulating kinetic trapping and thermodynamic stability.^3,4^ The extent and type of biopolymer assembly (Figures 1–3) modulates chemical lifetimes in ways that are not predicted by Δ*G*_(*r*)_^‡^ (*int*) (Figure 4).^3,4^ To describe this phenomena in general, we appropriated the term recalcitrance and define it as a general tendency of assembly to increase chemical lifetimes (persistence).^3,4^ The term recalcitrance is taken from carbohydrate chemists^37^ who use it to describe the resistance of polyglucose in crystalline cellulose to hydrolysis. Polyglucose in crystalline cellulose is completely unreactive, even to enzymes.^49^ The activation energies for essentially any chemical transformation of cellulose include the term – Δ*G* (*cryslallize*) (Figure 4C), meaning that the activation energy for a reaction includes the free energy of disassembly. Cellulose recalcitrance is a barrier to biofuel production.

Cellulose is not unique in its recalcitrance. All biopolymers access recalcitrant states. Fibrous proteins and amyloids hydrolyze more slowly and are more persistent than globular domains.^50,51^ Disordered linker regions between globular domains hydrolyze more readily than globular domains.^52,53^ Assembled collagen is so recalcitrant it has been detected in dinosaur fossils.^54,55^ Single-stranded DNA is more vulnerable to chemical and nucleolytic degradation than double-strand DNA.^43,56,57^ Folded tRNAs and rRNAs are persistent and robust (Figure 3b), whereas unfolded mRNAs are labile and short-lived.^3^ Polyglucose can persist for hundreds of millions of years,^58^ or not,^38^ depending on its assembly state. Biopolymers fall on a continuum; some biopolymers maintain reduced reactivity in assemblies^59−61^ while others are essentially unreactive in assemblies.

### Goldilocks Recalcitrance

**Figure 5:**
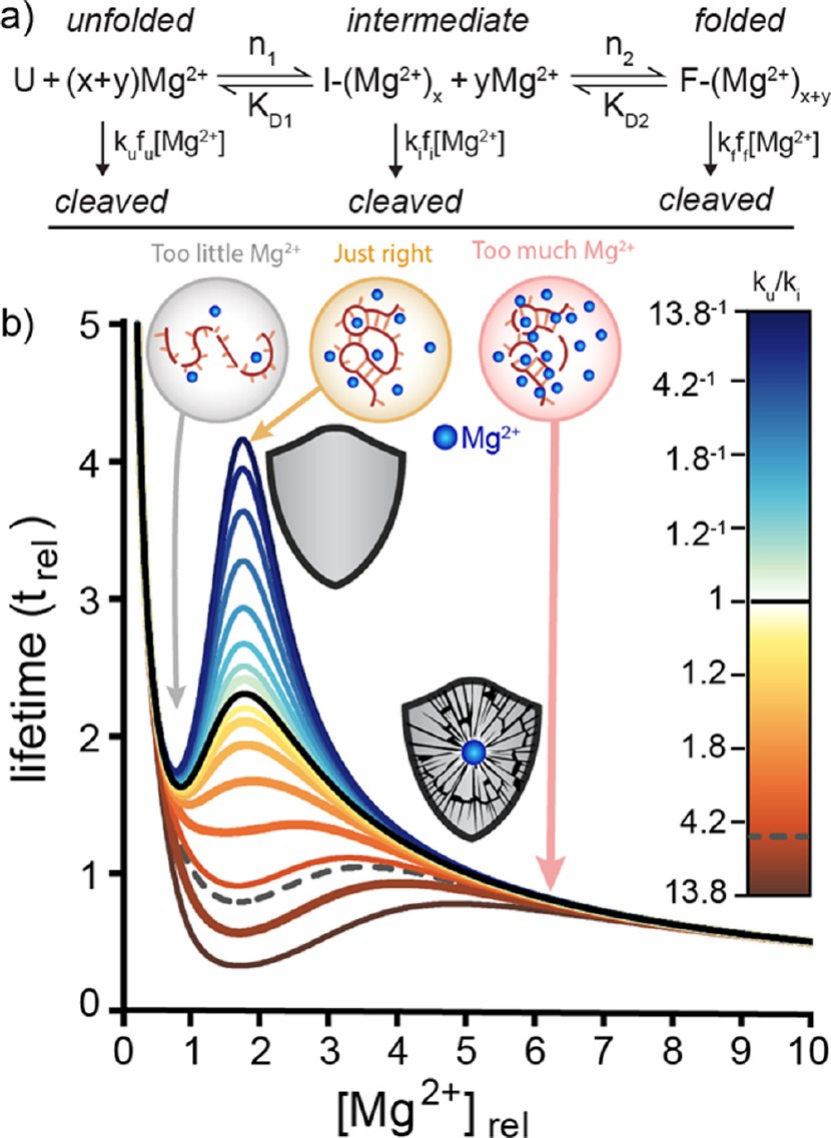
Simulation of RNA recalcitrance shows Goldilocks peaks of protection. (a) Unfolded RNA converts by one transition to an intermediate and by a second transition to a fully folded state with increasing [Mg^2+^]. Unfolded RNA is cleaved with a rate constant *k*_u_, the intermediate is cleaved with a rate constant *k*_i_, and the fully folded state is cleaved with a rate constant of *k*_f_. (b) In this simulation, *k*_i_/*k*_u_ was varied while other parameters were fixed. The black line represents lifetimes when *k*_i_ = *k*_f_. The dashed line represents the lifetimes when *k*_i_ = *k*_u_. The most pronounced Goldilocks peak is observed when *k*_i_ < *k*_f_. The color bar on the RH side indicates *k*_i_/*k*_u_. Adapted from ref (3). Available under a CC-BY 4.0 international license, copyright Loren Williams.

Nucleic Acids are incredibly sophisticated in that they appear to have the greatest range and control of persistence. Intrinsically, RNA is especially labile,^62^ meaning that Δ*G*_(*r*)_^‡^ (*int*) (Figure 4) is less for RNA than for other biopolymers. Self-cleavage of RNA involves nucleophilic attack of the 2′-oxygen of the ribose on the adjacent phosphorus atom. The reactivities of 2′-oxygens and the chemical lifetime of RNA are modulated by folding. Using simulation and experiment we validated a Goldilocks model of RNA recalcitrance (Figure 5).^3^ As experimental models we used yeast-tRNA^Phe^, the *Tetrahymena* ribozyme P4–P6 domain and polyU_20_ (polyuridylic acid 20-mer). For RNAs that fold, local maxima in lifetime are surrounded by conditions of greater lability. For example, RNAs can resist cleavage under conditions where Mg^2+^ folds the RNA. Increasing [Mg^2+^] beyond the folding threshold or decreasing to less than the folding threshold increases rates of cleavage. Goldilocks regions were observed when RNA was ∼95% folded, whereas a control RNA that does not fold, rU_20_, did not display Goldilocks behavior. Goldilocks recalcitrance explains how lifetime landscapes are modulated by specific characteristics of RNAs and by conditions related to monovalent and divalent cation concentrations, ligand concentrations, water activity, and temperature. RNAs that do not fold, do not access Goldilocks self-protection. Self-cleaving ribozymes are exempt from Goldilocks behavior because their folding increases rates of cleavage. We propose that Goldilocks recalcitrance was a selectable trait of biopolymers during pre-Darwinian evolution

### Heterorecalcitrance

Biopolymers can shelter and protect each other. Nucleic acids are recalcitrant when bound by proteins. Hetero-recalcitrance is the basis of enzymatic and chemical footprinting of DNA–protein or RNA-protein complexes.^63−65^ Because of hetero-recalcitrance, interactions between nucleic acids and proteins can be mapped with reactive chemical probes, including hydroxyl radical, dimethyl sulfate, and lead acetate. Regions of nucleic acids that interact with protein are more recalcitrant (less reactive) than unbound regions. We support a model in which hetero-recalcitrance was an important mechanism of coevolution of biopolymers in the evolutionary lead-up to Darwinian processes.

### Recalcitrance and Evolution

Biological systems display incredible control of chemical reactivities and can manipulate both the activation energies and net free energies of any given reaction, in isolation of all other reactions. Enzymes stabilize transition states and decrease activation energies by ΔΔ*G*_(*f*)_^‡^ (*enz*). In contrast to enzymes, recalcitrance can decrease a reaction rate in one direction without affecting the rate in the reverse direction (Figure 4). Recalcitrance increases thermodynamic stability and modulates reactivity in one direction only. Cellulose is an extreme but is not an anomalous example of recalcitrance. For this system the assembled state is completely unreactive; ΔΔ*G* (*rec*) is equivalent to the free energy of assembly. The general proficiency for control of chemical reactivity by biopolymers allows us to recognize them as products of evolution, and not products of nonevolutionary physical, chemical, or geological processes.

## Mutualisms

We argue that evolutionary concepts can have significant explanatory utility in chemistry and biochemistry, offering frameworks to understand structures, functions, and origins of molecules. Mutualisms illustrate this power. Formalisms developed by biologists to describe mutualistic relationships at cellular, organismal, and ecosystem levels can also elucidate cooperative interactions among biopolymers and other biological molecules. By viewing molecules as participants in mutualistic networks, we can learn about chemical and biological complexity, and emergence and evolution.^66^

**Figure 6:**
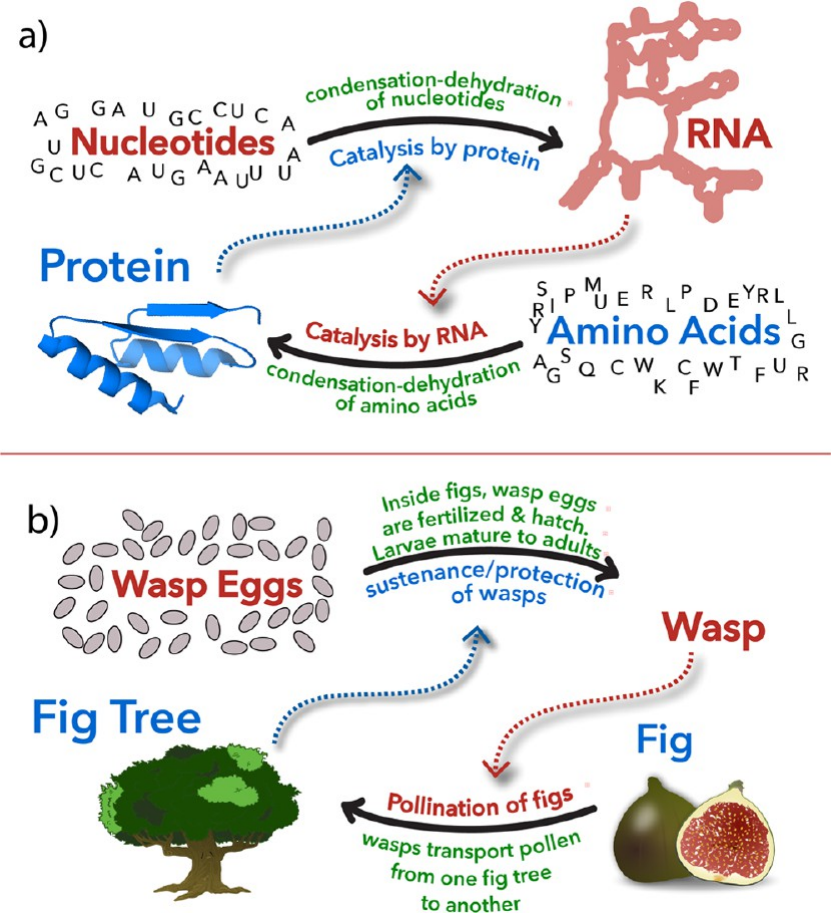
Mutualisms benefit partnering species. (a) Molecular mutualism. Proteins make RNA, and RNA makes protein. (b) The fig–wasp mutualism. The fig depends on the wasps to pollinate fig flowers and initiate seed production. The wasp depends on the fig for nourishment and production of offspring. Adapted from ref (66). Available under a CC-BY 4.0 international license, copyright Loren Williams.

A mutualism (Figure 6) is a persistent and intimate interaction that benefits partnering species.^67,68^ A mutualism is reciprocal exchange; a species proficient in obtaining certain benefits confers those on a second species, which reciprocates by conferring different benefits on the first species.^69^ Mutualisms are everywhere in the biosphere and are fundamentally important in ecology.^70^ All species on Earth participate in mutualisms. Mutualisms can increase productivity, abundance, and temporal stability of both mutualists and nonmutualists in food webs.^71^ Mutualisms (i) sponsor coevolution, (ii) foster innovation, (iii) increase fitness, (iv) inspire robustness, (iv) are resilient and resistant to change, and (v) involve partners that are distantly related with contrasting yet complementary proficiencies.

Mutualisms were previously understood to operate on levels of cells, organisms, ecosystems and even societies and economies. The eukaryotic cell is a culmination of mutualism between simpler prokaryotic cells.^72−74^ The majority of land plant families are mycorrhizal. This plant-fungi mutualism is traceable to the origins of land plants.^75^ Flowering plants such as the fig (Ficus spp., Moraceae) and insects such as the fig wasp (Agaonidae, Chalcidoidea) form obligate mutual relationships (Figure 6B).^76^ The wasp depends on the fig for food and the fig depends on the wasp for pollination. Pollen-bearing female wasps initiate seed production in the fig by delivering pollen. The fig provides each wasp larva with a fig seed, which is consumed by the wasp.

The formalisms describing mutualisms on levels of cells, organisms, and ecosystems apply equally well to molecules.^66^ For example, biopolymers are synthetically interdependent. RNA synthesizes protein in the ribosome and protein synthesizes RNA in polymerases. Mutualisms describe heterorecalcitrance. By forming assemblies, biopolymers protect each other from chemical assault. Proteins and peptides promote folding and functions of RNA^77−82^ and vice versa.^83,84^ Mutualisms describe protein-based pores and pumps in bilayer compartments.^85^ A cell can be understood as a consortia of molecules in mutualism relationships; an Amazon Jungle of molecules (described by the interactome). Mutualisms drive coevolution, thereby resolving ’chicken and egg dilemmas’^86^ in the chronology of RNA and protein origins.

Molecular mutualisms can also be manifested as covalent linkages between different classes of biopolymers. For example, polyglycans covalently linked to proteins comprise 50% or more of the total molecular weight of a glycoprotein. Protein glycosylation, which is a result of cotranslational or posttranslational modification, affects protein solubility, folding, and aggregation. Lipidation of peptides and proteins with long-chain lipids, which is a common endogenous post-translational modification in today’s biology, has been shown to induce membrane association. Lipidation can modify the biophysical properties of the covalently linked peptides, including their water solubility, self-aggregation propensity, and thermal stability.

### Molecular Mutualisms in the Origins of Life

In evolutionary models of proposed here, molecular mutualisms predate biopolymers. In these models, mutualisms were important among molecular ancestors of DNA, RNA, protein and polysaccharides, providing mechanisms of biopolymer coevolution. Mutualisms between molecules in a prebiotic environment would have expanded the chemical landscape and the space for chemical selection. We hypothesize that ancestral mutualisms involved heterorecalcitrance, chaperoning of folding or solubility, catalysis and autocatalytic cycles.

We have experimentally confirmed mutualisms between RNA and proto-peptides (polyesters and depsipeptides), which form easily in dry-down reactions. Depsipeptides contain backbone ester linkages in place of some amide bonds, and are proposed to be the ancestors of peptides.^87,88^ Depsipeptides form readily under mild dry-down of mixtures of hydroxy acids and amino acids.^87,89−93^ Ester linkages enable the formation of amide bonds through a process of ester–amide exchange.^87,90,91^ We have observed that this catalytic conversion of esters to amides is not reversible under the conditions of the experiment due to kinetic trapping. This lack of reversibility suggests a special relationship between activation energies, free energies of reaction, and temperature. Such special relationships are expected from evolutionary processes.

**Figure 7:**
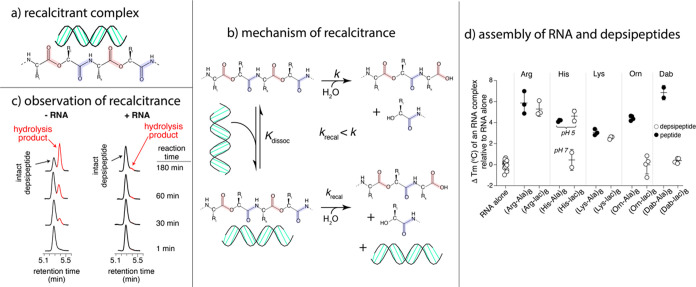
Heterorecalcitrance and molecular mutualism in a model prebiotic system. (a) A schematic diagram of a complex of a cationic depsipeptide and an RNA duplex. (b) A kinetic model of heterorecalcitrance in which the rate of hydrolysis of a depsipeptide is reduced by association with RNA. (c) An experimental demonstration of heterorecalcitrance showing that the rate of hydrolysis of a depsipeptide is reduced by association with an RNA duplex. This image shows HPLC traces (270 nm) of intact and cleaved depsipeptides at various time points in the presence or absence of the RNA duplex at 37C. (d) Association with cationic depsipeptides increases the stability of the RNA duplex to thermal melding. The RNA duplex is (5′-rCrGrCrUrArArArUrCrG-3′ and 5′-rCrGrArUrUrUrArGrCrG-3′, 2.5 uM strand). The depsipeptides (100 uM) are in buffered solution (10 mM phosphate, 100 mM NaCl, pH 7.0 or 10 mM acetate). Ac (acetyl) or Aba (acetamidobenzoic acid) was appended to the N-termini to increase UV absorbance. Adapted from ref (1). Available under a CC-BY 4.0 international license, copyright Loren Williams.

Our molecular mutualism experiments show that cationic depsipeptides interact with RNA duplexes and stabilize them^1^ (Figure 7). Various cationic depsipeptides increase the Tm of RNA duplex melting. Depsipeptides containing positively charged proteinaceous amino acids (Lys, Arg, or His) promote RNA duplex stability to a greater extent than depsipeptides containing nonproteinaceous prebiotic building blocks (ornithine, 2,4-diaminobutyric acid, or 2,3-diaminopropionic acid). The ineffectiveness of depsipeptides containing ornithine and 2,4-diaminobutyric acid in increasing RNA thermal stability is attributed to more facile intramolecular O,N-acyl transfer reactions in these structures compared to the positively charged proteinaceous amino acids (Arg, Lys, or His), leading to the degradation of ornithine- and 2,4-diaminobutyric acid-containing sequences during thermal melting. RNA in turn can stabilize and extend the chemical lifetimes of cationic depsipeptides. Specifically, association with an RNA duplex increased the observed lifetime of a depsipeptide by up to ∼30-fold. A single strand of RNA increased the depsipeptide lifetime, but to a lesser extent (about 5-fold). These results, combined, are a demonstration of the possibility of primitive mutualisms between proto-biopolymers, where both gain fitness by association.

α-Hydroxy acids can be incorporated ribosomally during translation to generate depsipeptides and polyesters, supporting the notion that depsipeptide and polyester could have been primordial versions of today’s proteins.^94,95^ Hydroxy acids are produced together with amino acids in model prebiotic reactions,^96^ are found together in some meteorites,^96,97^ and can combine to form oligomers >20 residues in length in mild dry-down reaction conditions.^87,89−93^

## Emergence

Evolution is creative.^98^ To paraphrase Dobzbansky:^99^*Evolution is a creative adventure*. *It is creative in the sense that an artist is creative*. *It brings about absolute novelties, constellations of genes [and molecules] which did not exist anywhere before*. *Evolutionary creativity, as artistic creativity, involves a risk of failure, miscreation, which in the biological world means death, extinction*. As noted by Maynard Smith, creativity in biology is hierarchical and chronological.^100^ As noted by Jacob, biochemical creativity occurred early, before LUCA.^101^ Metabolic creativity was next,^102^ followed by multicellularity.^103^ Creativity in neurology is ongoing.^104^

Evolution gives rise to emergence.^105^ The products of evolution are always interdependent multicomponent systems that exhibit emergence, where system properties differ fundamentally from the properties of isolated system components.^106^ Emergence can be envisioned as passage through a metaphorical door; when a system transitions to a new emergent state, new rules materialize. Emergence gives rise to complex functions that are not evident in the isolated parts of the system. The ribosome, the spliceosome, and the mitochondrion are creative inventions of evolution that demonstrate emergence. The ribosome, the spliceosome, and the mitochondrion stand as witness to the power of evolution to foster emergence.

**Figure 8:**
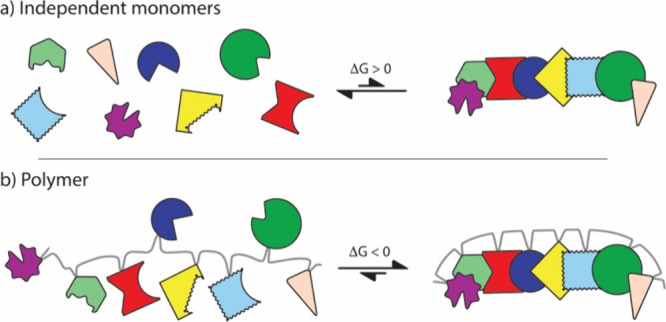
Biological assemblies are emergent on polymerization. Emergence gives rise to new behaviors. A solution of diverse small molecules will not crystallize or otherwise assemble via specific interactions. However, if the small molecules are polymerized, especially in specific sequences, they spontaneously assemble, for example, by folding. The colored shapes represent biopolymer building blocks. The gray line represents the biopolymer backbone.

Each biopolymer is an emergent molecule. The structures, functions and properties of biopolymers are different from those of the monomeric building blocks. Monomeric amino acids do not self-assemble into enzymes, fibers, compartments, or motors (Figure 1). Those assemblies are emergent on polymerization (Figure 8). Similarly, the structures and functions of polysaccharides (Figure 2) cannot be achieved by monomeric sugars, as glucose alone does not form fibers, helices, or dendrites. The same holds true for RNA; monomeric nucleotides in aqueous solutions do not spontaneously form base pairs.^107^ Each type of biopolymer behaves differently from its nonpolymerized constituents, consistent with predictions of creation through evolution. The emergent properties of biopolymers are evidence for their creation via evolutionary processes.

It has been said that evolution can give *the appearance* of design.^108^ Evolution creates complexity, functionality and emergent phenomena that naively seem to be designed for purpose.^109^ Such appearance does not mean that evolution acts with intentionality or foresight; it does not. Evolution has no more consciousness or intelligence or foresight than do gravity or electromagnetism.^108^

## Darwinian and Non-Darwinian Evolution

Biopolymer backbones share many attributes with each other and are imprinted with the footprints of evolution (Figures 1 and 2). Biopolymers are fragile but are protected by recalcitrance and are wildly abundant over the Earth. They engage in intense mutualisms. Their functions are transformed by subtle chemical changes. Yet each type of biopolymer is structurally and functionally distinct from the others. The totality of biopolymer proficiencies is far greater than the sum of their parts. Structures and functions of biopolymers in combination are emergent and cannot be recapitulated with isolated biopolymer types. Replication requires both a protein polymerase and nucleic acid template. A simple model to account for the emergent properties of biopolymers is their creation via coevolution in a common milieu in which control via homo and hetero-recalcitrance over hydrolytic degradation and other chemical assaults was a unifying early selective principle.^4^ Nonbiological species such as polypropylene and quartz are technologically useful but do not exhibit emergence, are not created by evolution, and therefore are readily distinguishable from biopolymers.

The evolutionary origins of biopolymer backbones seem undeniable. Yet, these backbones remain fixed and invariant throughout all known Darwinian evolution. Nowhere in the vast and diverse tree of life do we find ribosomes made from anything other than RNA and protein. The dependence of Darwinian evolution on evolved biopolymer backbones, combined with its inability to evolve them, presents a critical paradox.

Here we seek a resolution of this paradox. Where did biopolymer backbones come from? Is there a defensible model that can explain and predict a creative progression from simple molecules of prebiotic chemistry to complex biopolymers? Can we envision and recapitulate a manner of non-Darwinian evolution that could produce biopolymers? We believe the answer will ultimately be yes.

Although we do not yet have a mature and fully functioning model of chemical evolution, we have described an experimental system and theoretical model by which ancestors of biopolymers might have arisen by non-Darwinian evolutionary processes.^5,110^ This model integrates chemical sciences and evolutionary theory; chemical evolution transitions seamlessly into Darwinian evolution. Our working definition of chemical evolution is continuous chemical change with exploration of new chemical spaces and avoidance of equilibrium.^5^ We propose that large and diverse populations of small molecules, proto-oligomers and proto-biopolymers were iteratively and recursively selected and sculpted and exapted to produce the building blocks and recalcitrant biopolymers that enabled Darwinian evolution, and survive in extant biology. Chemical evolution is sustained by a flux of molecules through iterative filters that select molecules that alter the filters. For example, the production of peptides enables assemblies that decrease rates of hydrolysis of peptides and other molecules that associate with peptides.

To follow evolution of complex mixtures during wet–dry cycling, we investigated changes over wet–dry cycles of a mixture containing 9 components. Analysis of reaction products was monitored by HPLC, NMR, and LC-MS.^5^ The rate of chemical change was greatest in early cycles, then declined, and stabilized at a nonzero value for the duration of the cycling. The data are consistent with a model in which the system continuously evolved and did not converge, or reach a steady state, throughout the course of the experiment. We have not yet experimentally determined whether prolonged chemical evolution avoids degeneration into steady state. Avoidance of steady state may require feeding and/or complex types of thermodynamic cycling (day/night plus seasons plus random weather, etc.).

Our evolutionary model maps elements of biological evolution onto chemical processes. We say that during environmental wet–dry cycling: (a) a generation is a single cycle; (b) heredity is information passed from one generation to the next; (c) information is associated with nonrandom chemical composition; (d) selection is preferential inheritance of certain molecular compositions; (e) fitness is persistence of molecules and specific molecular assemblies; (f) variation is spatiotemporal differences in information; (g) an individual is a chemically isolated molecular ensemble; and (h) water is the “energy currency” that thermodynamically links molecules to each other and to the environment. During the origins of life, a “system” harvested energy from the “surroundings” and invested it in creating biopolymers. In this model biological molecules are products of evolution and are not necessarily represented in abiotic inventories on the ancient earth. Chemical evolution does not require biological molecules or template-directed replication.

In sum, we present a model, and certain data to support it, in which life on Earth was preceded by, and sponsored by, sustained chemical evolution. We propose that the chemical evolutionary process that led to biology is a special case of a general phenomenon. Chemical evolution, once understood, might have the potential to transform chemical sciences in general. This model opens the exciting possibility of applications of directed chemical evolution to a broad range of applications ranging from pharmaceuticals to material sciences. If an evolutionary process produced incredible molecules such as RNA and protein, then humankind can gain advantage by understanding and redirecting that process.

## Models and Data

Models of direct chemical synthesis of biopolymers have dominated origins of life research over the last half century. In these nonevolutionary models, extant building blocks, or their close chemical analogs, arose^111^ and polymerized via direct synthetic chemistry on the abiotic Hadean Earth.^86,112−114^ These nonevolutionary models assume that combinations of fortuitous geologic, organic and inorganic processes produced biopolymers, which have remained fixed over all of evolution.

The essence of these models was expressed in a recent review,^115^ which states, “···the core structure of nucleic acids appears to be a natural outcome of non-biological chemical processes···approximately 4.36 ± 0.05 billion years ago.” In these direct synthesis models, biology incorporated and has maintained prebiotic building blocks and polymers; extant biopolymers provide information on prebiotic chemistry. As noted in a second review,^114^ “···extant life, despite billions of years of evolution, has retained some direct vestiges of its prebiotic chemistry.”

These conventional models generally assume that all evolution is Darwinian. The assumption of a single kind of evolution is the basis of RNA World models. “···Darwinian evolution is the only mechanism by which matter can organize itself to give properties that we value in life.”^115^

By contrast, in the evolutionary model proposed here, evolution has evolved. Chemical species that arose via direct synthetic processes on the Hadean Earth were sculpted, selected, exapted, resculpted, reselected, and re-exapted during creative chemical coevolutionary processes. In this process, biopolymer backbones, were selected for polyfunction (Figures 1 and 2). We support a model of coevolution of biopolymer backbones, the inventories of amino acids, nucleotides and sugars, the genetic code, and energy currency and metabolism. In this evolutionary model, the link between prebiotic chemistry and biochemistry is lost.

Our conclusion that biopolymer backbones are evolutionary products suggests that ancestors of extant backbones once existed but are now extinct. This extinction model is consistent with the architecture of the ancient ribosomal core, which appears to retain information about extinct backbones. Our previous interpretation of ribosomal structures^14,116,117^ is that diverse ancestors of coded proteins, synthesized before coding emerged and before the subunit interface formed, interacted with RNA ancestors via complementary surfaces. These ancestral species were eventually replaced by modern biopolymers, preserving ancestral conformations and molecular interactions within the modern day ribosome.

The evidence that biopolymers are products of chemical evolution is independent of our lack of complete understanding of mechanisms of chemical evolution. The strong evidence for biopolymer evolution cannot be discounted simply because we do not fully understand mechanisms of that evolution. Historically, distinction between data and models is illustrated by the rejection of strong evidence of plate tectonics by many geologists in the early and mid 20th century in part because they could not imagine a model for movement of continents.^118^ The evidence for biopolymer evolution is sufficiently strong that Darwinian evolution should be discounted as the sole mechanism by which matter can organize and evolve.

Evolutionary models of biopolymer origins are departures from previous models of direct chemical synthesis. Evolutionary models are consistent with Orgel’s “gloomy” prediction^119^ that biochemistry lost vestiges of prebiotic chemistry. Chemical evolution may have substantially erased and rewritten prior prebiotic chemistry. If so, how do we confront the origins of life? What experiments should we do? In fact, evolutionary models of biopolymer origins are experimentally accessible, for example by wet–dry or freeze–thaw cycling. There is much to be learned about effects of duration, feeding, seeding, library composition, cycling temperature and frequency, low frequency perturbations (seasons), etc. A lack of direct connection of biochemistry to prebiotic chemistry should not deter us from constructing and experimentally testing evolutionary models. Currently we do not know if it is possible to recapitulate and control specific steps in chemical evolution as it occurred on the early Earth. Human labor probably cannot do what evolution can do. We can hope to someday understand what evolution has done and influence what evolution will do. We believe that new models integrating evolutionary theory into chemical sciences will lead to advances in prebiotic chemistry and in chemical sciences in general. A change of paradigm seems positive and exciting.

Our goal is to resolve the paradox of the evolutionary origins of biopolymer backbones and the absolute dependence of Darwinian evolution on the invariance of biopolymer backbones. We extend Dobzhansky “Nothing in biology makes sense, except in light of evolution”,^120^ to molecules and argue that nothing in biochemistry makes sense, except in light of chemical evolution (also see^8^).
